# Efficacy and safety of nintedanib and docetaxel in patients with previously treated lung non-squamous non-small cell lung cancer: a multicenter retrospective real-world analysis

**DOI:** 10.2478/raon-2023-0040

**Published:** 2023-09-04

**Authors:** Lidija Ljubicic, Urska Janzic, Mojca Unk, Ana Sophie Terglav, Katja Mohorcic, Fran Seiwerth, Lela Bitar, Sonja Badovinac, Sanja Plestina, Marta Korsic, Suzana Kukulj, Miroslav Samarzija, Marko Jakopovic

**Affiliations:** Department for Respiratory Diseases Jordanovac, University Hospital Centre Zagreb, Zagreb, Croatia; Medical Oncology Unit, University Clinic Golnik, Golnik, Slovenia; Faculty of Medicine, University of Ljubljana, Ljubljana, Slovenia; Division of Medical Oncology, Institute of Oncology Ljubljana, Ljubljana, Slovenia; School of Medicine, University of Zagreb, Zagreb, Croatia; School of Medicine, University of Rijeka, Rijeka, Croatia

**Keywords:** advanced NSCLC, antiangiogenic therapy, docetaxel, nintedanib, real-world data

## Abstract

**Background:**

The standard first-line systemic treatment for patients with non-oncogene addicted advanced nonsquamous non-small cell lung cancer (NSCLC) is immunotherapy with immune checkpoint inhibitors (ICI) and/or chemotherapy (ChT). Therapy after failing ICI +/− ChT remains an open question, and docetaxel plus nintedanib represent a valid second line option.

**Patients and methods:**

A multicenter retrospective trial of real-life treatment patterns and outcomes of patients with advanced lung adenocarcinoma treated with docetaxel plus nintedanib after the failure of ICI and/or ChT was performed. Patients from 2 Slovenian and 1 Croatian oncological center treated between June 2014 and August 2022 were enrolled. We assessed objective response (ORR), disease control rate (DCR), median progression free survival (PFS), median overall survival (OS), and safety profile of treatment.

**Results:**

There were 96 patients included in the analysis, with ORR of 18.8%, DCR of 57.3%, median PFS of 3.0 months (95% CI: 3.0–5.0 months), and a median OS of 8.0 months (95% CI: 7.0–10.0 months). The majority of patients (n = 47,49%) received docetaxel plus nintedanib as third-line therapy. The ORR for this subset of patients was 19.1%, with a DCR of 57.4%. The highest response rate was observed in patients who received second-line docetaxel plus nintedanib after first-line combination of ChT-ICI therapy (n = 24), with an ORR of 29.2% and DCR of 66.7% and median PFS of 4.0 months (95% CI: 3.0–8.0 months). Fifty-three patients (55.2%) experienced adverse events (AEs), most frequently gastrointestinal; diarrhea (n = 29, 30.2%), and increased liver enzyme levels (n = 17, 17.7%).

**Conclusions:**

The combination of docetaxel and nintedanib can be considered an effective therapy option with an acceptable toxicity profile for patients with advanced NSCLC after the failure of ICI +/− ChT.

## Introduction

Lung cancer remains the leading cause of cancer death, with an estimated 1.8 million deaths worldwide in 2020.^1^ With the identification of oncogene drivers in non-small cell lung cancer (NSCLC), the prognosis of patients harboring specific alterations has dramatically improved. However, the proportion of these patients remains low, the prevalence of targetable alterations depends on many factors, and drug resistance presents an unavoidable fact that limits the efficacy and the use of targeted drugs. For non-targetable advanced NSCLC, limited treatment options lead to worse outcomes.^2^ Therefore, more therapeutic options are needed for both groups of patients with advanced NSCLC, those with driver mutations, and others without, after progression on either targeted therapy, checkpoint inhibitors (ICI) alone, or in combination with chemotherapy (ChT). Nowadays, the complexity of the tumor microenvironment is increasingly emphasized because it abounds with various pro-angiogenic factors such as vascular endothelial growth factor (VEGF), basic fibroblast growth factor (bFGF), and platelet-derived growth factor (PDGF).^3^ Angiogenesis is crucial for tumor growth, maintenance, and metastasis.^4^ The concept of antiangiogenic therapy is evolving and gaining attention due to its essential role in tumor development. Despite initial high expectations, antiangiogenic monotherapies have shown only modest clinical benefit, primarily due to the development of resistance. Several different mechanisms are involved, such as vessel co-option, vasculogenic mimicry, and activation of other substitute pathways.^5,6^ The combination of antiangiogenic therapy with different therapeutic strategies could overcome resistance.^7^

Currently, several antiangiogenic therapies are available for the treatment of different tumor types, most of which target the VEGF signaling pathway. Bevacizumab was the first Food and Drug Administration (FDA) angiogenesis inhibitor approved in 2006 for NSCLC in combination with chemotherapy for the treatment of patients with advanced non-squamous NSCLC.^8^ Ramucirumab and nintedanib are two other FDA, and European Medicines Agency (EMA) approved antiangiogenic agents for the treatment of an advanced NSCLC. In 2014, EMA approved nintedanib plus docetaxel for the treatment of patients with advanced lung adenocarcinoma following first-line ChT based on the results of LUME-Lung 1 (phase III trial), which enrolled 1,314 patients with advanced or recurrent NSCLC. In combination with docetaxel, nintedanib proved to be more effective than docetaxel alone in delaying cancer progression with median progression free survival (mPFS) of 3.5 months in the overall study population receiving docetaxel plus nintedanib, compared with 2.7 months in patients receiving docetaxel alone.^9^

While the efficacy and safety of docetaxel plus nintedanib has already been confirmed in clinical trials, we aim to provide insight into whether real-world data are comparable to those from clinical trials. We also compared the safety and tolerability of this combination with results found in the current state-of-the-art literature.

## Patients and methods

This was a retrospective, non-interventional, multicenter, real-world analysis of patients with advanced/metastatic NSCLC with adenocarcinoma histology/cytology treated with a combination of docetaxel and nintedanib in different treatment lines between June 2014 and August 2022. Data were sourced from two Slovenian (University Clinic Golnik and Institute of Oncology Ljubljana, Slovenia) and one Croatian center (University Hospital Center Zagreb, Croatia). The study was performed in accordance with the Helsinki Declaration ethical standards for biomedical studies on humans and was approved by the Ethics Committee of University Hospital Center Zagreb (Decision number 02/013 AG).

Data collected from the patients’ medical records included the following: sex, age, European Clinical Oncology Group (ECOG) performance status (PS) before starting docetaxel and nintedanib combination, clinical stage based on the 8^th^ edition of the International Union Against Cancer and American Joint Committee on Cancer TNM Classification of Malignant Tumors, biomarker testing results (epidermal growth factor receptor (*EGFR*) mutation, anaplastic lymphoma kinase (*ALK*) rearrangements, ROS Proto-Oncogene 1 (*ROS1*) rearrangement*s*, Kirsten rat sarcoma viral oncogene homolog (KRAS), mesenchymal-epithelial transition factor (MET), ret proto-oncogene (RET), fibroblast growth factor receptors (FGFR) and programmed death-ligand 1 (PD-L1) expression, smoking history, prior therapy regimen (ChT and/or iICI, tyrosine kinase inhibitors [TKI], radiotherapy), presence of brain metastases (assessed with computerized tomography [CT] and/or magnetic resonance imaging [MRI]) and adverse events associated with the use of docetaxel plus nintedanib. The response was assessed according to the Response Evaluation Criteria in Solid Tumors (RECIST) version 1.1.^10^ Adverse events were assessed using the Common Terminology Criteria for Adverse Events (CTCAE) v4.0 criteria.^11^ The cut-off date for analyzes was December 2022.

We assessed progression-free survival, objective response rate, overall survival, and the safety profile of patients treated with docetaxel and nintedanib. PFS was defined as the time from the initiation of therapy to the time of the earliest progressive disease (PD) or study cut-off. Overall survival was assessed from the initiation of treatment until the date of death from any cause or study cut-off. A swimmer plot was applied to present the clinical outcome of patients with EGFR mutated patients. Kaplan–Meier method was used to assess the PFS and overall survival (OS). To test the difference in survival between patients with and without brain metastases and the occurrence of adverse events (AEs), the log-rank test was used.

Patients were treated and followed up as per the standard of care in a routine clinical setting in 3 centers. The response was assessed by enhanced CT until disease progression or intolerable toxicity.

Patients were treated routinely with docetaxel every 3 weeks and nintedanib 200 mg twice daily according to the summary of product characteristic (SmPC) approval. In case of adverse events, treatment was interrupted and continued at a lower dose according to the standard guidelines.

All results were obtained and plotted using R v. 3.6.2 (R Core Team, 2017).

## Results

Ninety-six patients were enrolled in this study, of whom 41 were female. The median age was 59.5 years, ranging between 39 and 75. Seventy-four (77.1%) patients were current or former smokers. The most common clinical stage was IV. At the start of treatment with docetaxel plus nintedanib, 11 patients (11.4%) had ECOG PS 0, 67 (69.8%) had ECOG PS 1, and 18 (18.7%) had ECOG PS 2. Demographic data of enrolled patients are presented in Table 1.

**TABLE 1.** Demographic and baseline characteristics of 96 patients treated with docetaxel plus nintedanib

**Variable**	**N = 96**
**Age, mean (years)**	59.5 (39–75)
**Sex**	
Male	55 (57.3%)
Female	41 (42.7%)
**ECOG performance status**	
0	35 (36.4%)
1	57 (59.4%)
2	4 (4.2%)
**Smoking status**	
Current smokers	56 (58.3%)
Never smokers	18 (18.8%)
Former smokers	18 (18.8%)
Unknown	4 (4.2%)
**Clinical stage at diagnosis**	
Stage ≤ IIIB	9 (9.4%)
Stage IIIC	1 (1.0%)
Stage IV	86 (89.6%)
**Brain metastases**	
Yes	18 (18.7%)
No	78 (81.3%)
**PD-L1 expression**	
0%	35 (36.5%)
1–49%	34 (35.4%)
≥ 50%	16 (16.7%)
Unknown	11 (11.5%)
**Biomarker testing**	
*EGFR* mutation positive	5 (5.2%)
*ALK* re arrangement present	0 (0.0%)
*ROS1* rearrangement present	0 (0.0%)
*KRAS* mutation present	7 (7.3%)
*MET* rearrangement present	1 (1.0%)
*RET* rearrangement present	3 (3.1%)
*FGF*R rearrangement present	1 (1.0%)
**Docetaxel plus nintedanib line**	
Second-line therapy after first line combination ChT-ICI	24 (25%)
Second-line therapy after first-line platinum-based ChT	7 (7.3%)
Third-line therapy after first-line ChT and second-line ICI	47 (49.0%)
Third-line therapy after first-line ICI and second-line ChT	13 (13.5%)
Fourth or later-lines	3 (3.1%)
Other¶	2 (2.1%)

ALK = anaplastic lymphoma kinase; ChT = chemotherapy; ECOG = Eastern Cooperative Oncology Group; EGFR = epidermal growth factor receptor; FGFR = fibroblast growth factor receptors; ICI = immune checkpoint inhibitor; KRAS = Kirsten ras oncogene homolog; MET = tyrosine-protein kinase Met; PD-L1 = programmed death-ligand 1; RET = Ret Proto-Oncogene; ROS1 = ROS Proto-Oncogene 1

Two patients received third-line docetaxel plus nintedanib after first-line combination ChT-ICI and second line targeted therapy (capmatinib or pralsetinib)

None of the 96 patients had *ALK* or *ROS1* rearrangements, five patients had an *EGFR* mutation, one patient had *MET* exon 14 skipping mutation, three patients had *RET* rearrangement, one FGFR rearrangement was present, and *KRAS* mutation testing was positive in 7 patients. Sixteen patients had tumor PD-L1 staining ≥ 50%.

**FIGURE 1.**
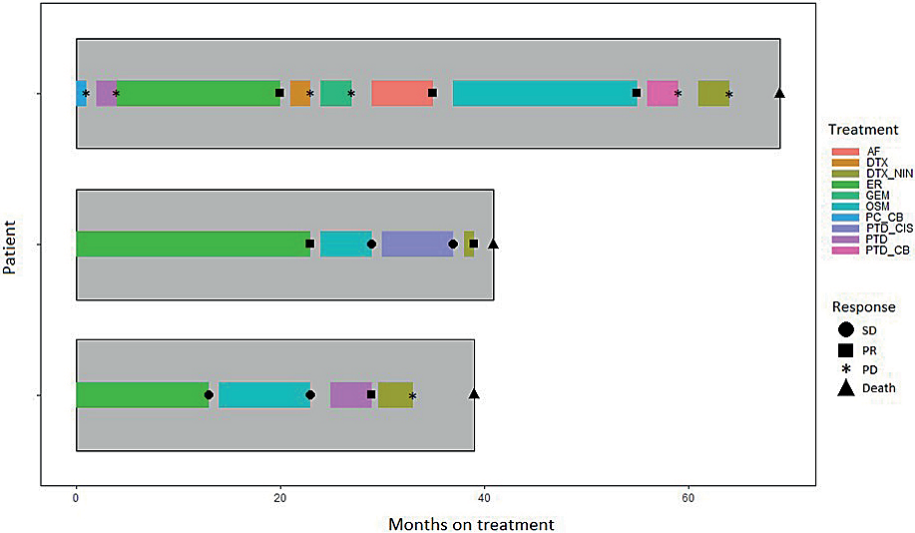
Swimmer plot of treatment duration and best treatment response in EGFR-positive patients. Different colours of the horizontal bars represent different treatment lines, while the symbols at the end of each bar represent the relevant responses. AF = Afatinib; DTX = Docetaxel; DTX_NIN = Docetaxel plus nintedanib; EGFR = epidermal growth factor receptor; ER = Erlotinib; GEM = Gemcitabine; OSM = Osimertinib; PC_CB = Paclitaxel and carboplatin; PD = progressive disease; PR = partial response; PTD_CIS = Pemetrexed and cisplatin; PTD = Pemetrexed; PTD_CB = Pemetrexed and carboplatin; SD = stable disease

The treatment sequences were as follows: 47 patients (49.0%) received docetaxel plus nintedanib as third-line therapy after first-line platinum-based ChT and second-line monotherapy with ICI, thirteen (13.5%) patients received docetaxel plus nintedanib as third-line therapy after first-line ICI monotherapy and second-line platinum-based ChT. Second-line docetaxel plus nintedanib was given to 24 patients (25%) after the first-line combination ChT-ICI therapy. Two patients received docetaxel plus nintedanib as third-line therapy after the first-line combination ChT-ICI therapy and after second-line targeted therapy (capmatinib or pralsetinib). A subset of seven patients received docetaxel plus nintedanib after a first-line platinum-based ChT. The remaining 3 patients (3.1%) received docetaxel plus nintedanib as a fourth-or later-line therapy. These were EGFR-positive patients who had received multiple lines of targeted therapy prior to docetaxel plus nintedanib (Figure 1).

The best response to treatment with docetaxel and nintedanib in all enrolled patients is presented in Table 2. 18 patients achieved partial response (PR), corresponding to objective response (ORR) of 18.8% (complete response [CR] was not observed), while 37 (38.5%) patients had stable disease (SD) and 31 (32.2%) patients had PD. The DCR (disease control rate) was 57.3%. Response to treatment with docetaxel and nintedanib for different treatment lines is presented in Table 3. Tumor response was not evaluable for 10 patients due to early treatment discontinuation or because the evaluation was not performed.

**TABLE 2.** Response to treatment with docetaxel plus nintedanib in all patients

**Tumor response according to RECIST version 1.1 criteria^10^**	**All patients** **N = 96**
CR	0 (0.0)
PR	18 (18.8)
SD	37 (38.5)
PD	31 (32.3)
ORR (CR+PR)	18 (18.8)
DCR (CR+PR+SD)	55 (57.3)
Non-evaluable	10 (10.4)
Median PFS, months	3.0 (95% CI: 3–5)
Median OS, months	8.0 (95% CI: 7–10)

CI = confidence interval; CR = complete response; DCR = disease control rate; ORR = objective response rate; OS = overall survival; PD = progressive disease; PFS = progression-free survival; PR = partial response; RECIST = response evaluation criteria in solid tumors; SD = stable disease

**TABLE 3.** Response to treatment with docetaxel plus nintedanib in different treatment patterns

**Tumor response according to RECIST version 1.1 criteria^7^**	**Second-line after a first-line combination ChT-ICI regimen (n = 24)**	**Second-line after a first-line platinum-based ChT (n = 7)**	**Third-line therapy following first-line ChT and second-line ICI (n = 47)**	**Third-line after first-line ICI and second-line ChT (n = 13)**	**Fourth or later-line treatment (n = 3)**
CR, n (%)	0 (0.0)	0 (0.0)	0 (0.0)	0 (0.0)	0 (0.0)
PR, n (%)	7 (29.2)	1 (14.3)	9 (19.1)	1 (7.7)	0 (0.0)
SD, n (%)	9 (37.5)	2 (28.6)	18 (38.3)	7 (53.8)	1(33.3)
PD, n (%)	3 (12.5)	3 (42.9)	18 (38.3)	5 (38.5)	2(66.7)
ORR, n (%)	7 (29.2)	1 (14.3)	9 (19.1)	1 (7.7)	0 (0.0)
DCR, n (%)	16 (66.7)	3 (42.9)	27 (57.4)	8 (61.5)	1(33.3)
Non-evaluable, n (%)	5 (20.8)	1 (14.3)	2 (4.3)	0 (0.0)	0 (0.0)

ChT = chemotherapy; CR = complete response; DCR = disease control rate; ICI = immune checkpoint inhibitor; ORR = objective response rate; PD = progressive disease; PR = partial reasponse; RECIST = response evaluation criteria in solid tumors; SD = stable disease

Two patients that received third-line docetaxel plus nintedanib after a first-line combination chemotherapy-ICI regimen and second-line targeted therapy (capmatinib or pralsetinib) are not listed in the table since it was not possible to evaluate the response to therapy.

**FIGURE 2.**
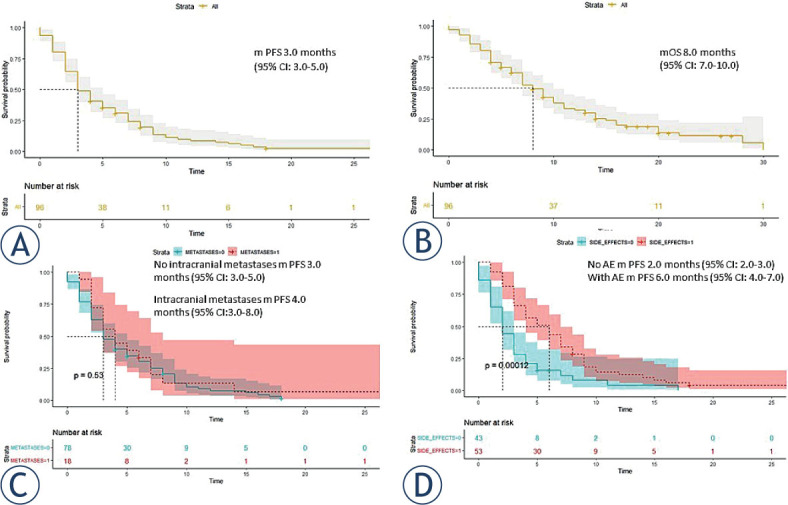
**(A)** Progression-free survival of all patients(PFS) (n = 96) treated with nintedanib and docetaxel combination therapy. **(B)** Overall survival (OS) of all patients treated with nintedanib and docetaxel combination therapy. **(C)** Progression-free survival (PFS) of patients with and without brain metastases. **(D)** Median progression-free survival of patients with and without adverse events.

At the data cut-off, median PFS (Figure 2A) and OS (Figure 2B) across all treatment lines (n = 96) were 3.0 months (95% CI: 3–5 months) and 8.0 months (95% CI: 7–10 months), respectively.

**FIGURE 3.**
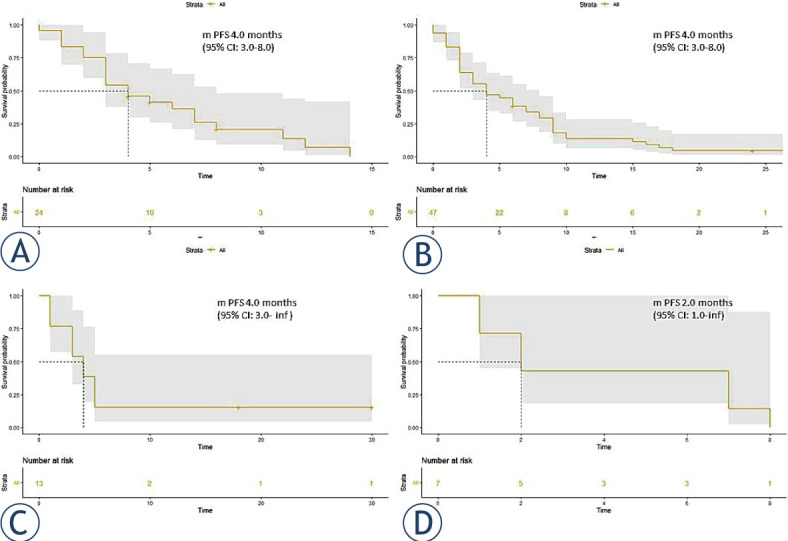
Outcomes with docetaxel and nintedanib across different treatment lines. Progression-free survival (PFS) of patients receiving docetaxel plus nintedanib as second-line treatment after first-line combination chemotherapy-checkpoint inhibitors (ChT-ICI) therapy **(A)**, third-line treatment after first-line platinum-based ChT and second-line ICI monotherapy **(B)**, third-line treatment after first-line ICI monotherapy and second-line platinum-based ChT **(C)**, and second-line treatment after first-line platinum-based ChT **(D)**.

The highest response rate was observed in patients who received docetaxel plus nintedanib as second-line therapy after first-line combination ChT-ICI therapy (n = 24), with an ORR of 29.2% and DCR of 66.7%. The median PFS for this subgroup of patients was 4.0 months (95% CI: 3.0–8.0 months) (Figure 3A).

For the subset of patients receiving docetaxel plus nintedanib as third-line therapy after first-line platinum-based ChT and second-line ICI monotherapy (n = 47), the observed ORR was 19.1% and DCR 57.4%. Median PFS was 4.0 months (95% CI:3.0–8.0 months) (Figure 3B). A similar efficacy was observed in a subset of patients receiving docetaxel plus nintedanib as third-line therapy after first-line ICI monotherapy and second-line platinum-based ChT with median PFS 4.0 months (95% CI: 3-inf) (Figure 3C).

The median progression-free survival was 3.0 months (95% CI: 3.0–5.0 months) for patients with no intracranial metastases and 4.0 months (95% CI:3.0–8.0 months) for patients with intracranial metastases (Figure 2C). However, there was no statistical difference in PFS between patients with and without brain metastases (*p* = 0.53).

## Safety of docetaxel plus nintedanib treatment

Table 3 gives the overview of adverse events (AEs) reported with docetaxel and nintedanib treatment. Fifty-three patients (55.2%) experienced treatment related AEs. The most common were gastrointestinal; diarrhea (n = 29, 30.2%) and elevated liver enzyme levels (n = 17, 17.7%), but mostly mild to moderate severity. Grade 3 AEs were observed in 8 patients (8.3%); 6 patients with elevated liver enzyme levels (6.3%), 1 patient with hypertension (1 %), and 1 with diarrhoea (1 %).

Other AEs reported were neutropenia (n = 4, 4.2%), stomatitis (n = 2), dermatitis (n = 6, 6.3%), nausea (n = 2, 2.1%), peripheral neuropathy (n = 3, 3.1%), and hypertension (n = 2, 2.1%) AEs were effectively managed by a dose reduction and did not require permanent discontinuation of treatment.

Thirty patients (31.2%) required temporary treatment discontinuation with docetaxel plus nintedanib. The main reasons were diarrhea (10.4%) and elevated liver enzymes (13.5%). Additional thirteen patients (13.5%) required a dose reduction of docetaxel mainly due to neutropenia and peripheral neuropathy, and eighteen patients (18.8%) required a dose reduction of nintedanib due to diarrhea and elevated alanine aminotransferase (ALT) and aspartate aminotransferase (AST) levels. Nineteen patients (19.8%) discontinued docetaxel plus nintedanib treatment due to AEs.

There was almost no difference in the frequency of AEs between the second-line and third-line docetaxel and nintedanib combination therapy (54.8% *vs.* 55%). Adverse events were more frequent (66.6%) in a subset of patients that received fourth-line docetaxel and nintedanib combination therapy. However, this finding is considered statistically insignificant due to the small sample size.

Patients who received immunotherapy before docetaxel and nintedanib had fewer adverse events than those not treated with immunotherapy.

**TABLE 4.** Differences in progression-free survival (PFS) and overall survival (OS) for each subset of patients according to the treatment line of docetaxel plus nintedanib

	**All patients (n = 96)**	**Second-line after a first-line combination ChT-ICI regimen (n = 24)**	**Second-line after a first-line platinum-based ChT (n = 7)**	**Third-line therapy after first-line ChT and second-line ICI (n = 47)**	**Third-line after first-line ICI and (n = 13) second-line ChT**	**Fourth- or later-lines treatment (n = 3)**
**Median** progression-free survival, months (95% CI)	**3** (3–5)	**4** (3–8)	**2** (1–inf)	**4** (3–8)	**4** (3–inf)	**3** (0–inf)
**Median** overall survival, months (95% CI)	**8** (7–10)	**9** (6–inf)	**10** (4–inf)	**10** (8–14)	**7** (3–inf)	**8** (2–inf)

Fewer than half of a group have experienced the event

ChT-ICI = chemotherapy-checkpoint inhibitors therapy; CI = confidence interval; inf = infinity

There were no treatment-related deaths due to AEs. In addition, characteristic AEs associated with VEGF pathway inhibition, such as arterial and venous thromboembolism, hemorrhage, and GI perforation, were not observed.

## Discussion

Previous studies have shown that the use of ICI with or without ChT as first-line therapy in patients with advanced NSCLC improves overall survival and progression-free survival.^12,13,14^ However, there is a lack of prospective, randomized controlled trials evaluating the optimal treatment for patients with advanced non-oncogene-addicted NSCLC after progression on ICI therapy with or without ChT. Despite the high initial efficacy of targeted therapies, drug resistance is inevitable, so finding new therapeutic options is also needed for patients who progress on targeted therapy. Chemotherapy has been considered as one of the standard treatments after acquiring resistance. Currently, available treatment options include single-agent chemotherapy combined with antiangiogenic drug such as nintedanib or ramucirumab.^15^

In our study, we aimed to demonstrate the multicenter experience and clinical characteristics of a cohort of patients with histologically confirmed advanced lung adenocarcinoma treated with docetaxel plus nintedanib in a real-world setting. Across all lines of treatment, median PFS was 3.0 months (95% CI: 3.0–5.0 months) and median OS 8.0 months (95% CI: 7.0–10.0 months). ORR was 18.8% and DCR was 57.3%. In a subset of patients receiving docetaxel plus nintedanib in the third-line setting, the ORR after first-line platinum-based ChT and second-line monotherapy with ICI was 19.1%. In comparison, the highest ORR (29.2%) was recorded in patients receiving docetaxel plus nintedanib as second-line therapy after first-line combination ChT-ICI therapy.

Approval of nintedanib in combination with docetaxel was based on the phase III LUME-Lung 1 trial.^9^ The addition of nintedanib to docetaxel significantly prolonged PFS in the entire study population, regardless of histology (3.4 versus *vs*. 2.7 months, HR 0.79; p = 0.0019). A significant improvement in median OS (from 10.3 to 12.6 months) was observed in patients with adenocarcinoma histology, particularly in those who progressed soon, within nine months after the start of first-line treatment (from 7.9 to 10.9 months).

A significant OS benefit in adenocarcinoma patients who progressed during or shortly after the end of first-line treatment was confirmed in a subanalysis of the adenocarcinoma population of the phase III LUME-Lung 1 trial (time from the start of first-line treatment < 6 months, mOS 9.5 (nintedanib/docetaxel) *vs.* 7.5 months (placebo/docetaxel) [HR 0.73, 95% CI 0.55–0.98)).^16^ A subanalysis of this trial also showed that the improvement in median OS with docetaxel plus nintedanib compared with docetaxel plus placebo was greater in the European adenocarcinoma population (4.7-month improvement in mOS).^16^

Over the past three years, several datasets about efficacy and tolerability of docetaxel plus nintedanib in the treatment of patients with advanced NSCLC after progression on platinum-based ChT followed by subsequent ICI treatment have been published. The most comprehensive retrospective real-world analysis was conducted by Metzenmacher *et al.*, and included 93 patients with NSCLC. In all evaluable patients, the ORR was 41.4%, and the DCR was 75.9%. The highest response rate was observed in patients who were treated with docetaxel plus nintedanib following the first-line ChT and second-line ICI (ORR of 50% and DCR of 82.7%). The median OS for this group was 8.4 months (95% CI: 5.0–11.0).^17^ Grohe *et al.* gave us an insight in a prospective VARGADO study by publishing the updated results for cohort B (n = 80), in which patients received docetaxel plus nintedanib after first-line ChT and second-line ICI therapy. In this study the median PFS was 6.4 months (95% CI: 4.8–7.3). At the time of analysis, the best ORR was 50% and DCR was 86%.^18^

**TABLE 5.** Overview of adverse events with docetaxel plus nintedanib treatment

Adverse Event^*^	**All grades n (%)**	**Grade 3 n (%)**
Total	53 (55.2)	8 (8.3)
Diarrhea	29(30.2)	1 (1.0)
Elevated liver enzymes	17(17.7)	6 (6.3)
Rash	6 (6.2)	
Neutropenia	4 (4.2)	
Peripheral neuropathy	3 (3.1)	
Stomatitis	2 (2.1)	
Nausea	2 (2.1)	
Hypertension	2 (2.1)	1 (1.0)

* Categorized according to the Common Terminology Criteria for Adverse Events (CTCAE) version 4.0

Corral *et al.* presented the results of their smaller cohort, which consisted of eleven patients. An ORR of 36.5%, DCR of 81.8%, and PFS of 3.2 were reported.^19^ Overall, we noted that the results presented in the above studies are consistent. In our subset of patients (n = 47) who received docetaxel plus nintedanib after first-line ChT and second-line ICI, response rates were lower (ORR of 19.1% and DCR of 57.4%). These outcomes, with lower DCR and PFS could have been due to the presence of poor prognostic factors of our patients included in the analysis (18.7% had brain metastases, 89.6% were found to have stage IV disease, and 64% were ECOG PS 1–2). However, more prospective studies are needed to verify these findings.

Eighteen patients included in our study already had evidence of intracranial disease progression. Most of our patients underwent whole brain radiation therapy (WBRT) due to multiple brain metastases, while in a smaller number of patients, gamma knife was performed. It is worth noting that no intracerebral complications were reported, and this group of patients responded as well to therapy as the others (Figure 2C).

Our analysis included five patients with *EGFR* mutations after failure to standard of care previous lines of therapy. Three of these patients received an EGFR-TKI before docetaxel plus nintedanib therapy. In two cases, a double mutation was found (coexistence of exon 19 deletion and exon 20 T790M). Patients received docetaxel plus nintedanib as fourth or later-line treatment. Two patients received EGFR-TKI as first-line treatment, while the remaining patient received a TKI as third-line therapy. An objective response rate and DCR were 0.0% and 33.3%, respectively. Although the LUME-Lung 1 trial did not evaluate *EGFR* mutation status, the efficacy of docetaxel and nintedanib in EGFR mutated NSCLC patients has been evaluated in recent clinical trials.^20,21^

Sixty-two patients were included in a study conducted by Hong *et al.* A median PFS of 6.5 *vs.* 3.3 months (EGFR mutated *vs.* EGFR not mutated) was considered promising, but further studies of the efficacy of docetaxel plus nintedanib in patients with EGFR-mutated NSCLC are needed.^21^

The toxicity profile was generally consistent with the known safety profile of this treatment combination, with diarrhea, elevated liver enzymes and rash beeing the most common adverse events.

Not all patients benefit from docetaxel plus nintedanib therapy, but there are currently no predictive biomarkers of response to antiangiogenic treatment. Our study demonstrated that the occurrence of AEs was associated with favourable efficacy in patients treated with this combination therapy. Median survival was two months in patients without any AEs and six months for patients with AEs. Several studies have demonstrated a correlation between the development of hypertension and longer PFS and/or OS in patients treated with antiangiogenic agents.^22,23^ In contrast, data are not yet available for combination therapy with docetaxel and nintedanib. However, the correlation between therapeutic efficacy and the occurrence of AEs remains unclear.

Our study has several limitations. The first limitation is the non-comparative, retrospective design. Another limitation is radiologic evaluation; RECIST measurements were not done by an independent radiologic review board but were performed during everyday clinical practice by a radiologist on duty. This could have led to non-homogeneous reviews with differences regarding target and non-target lesions. Because of the retrospective nature of data collection, underreporting of potential side effects may have occurred. Finally, due to the heterogeneity of the population under study (*i.e.*, different treatment lines), statistical power is decreased, resulting in nonsignificant differences between treatment groups in terms of outcome.

Our data support the use of docetaxel and nintedanib, which proved safe in 2nd and later lines, even in patients with previously treated brain metastases.

The benefit observed in ICI-pretreated patients is notable, and should be explored further to elucidate a synergistic effect between antiangiogenics and ICI.

In addition, further studies are needed to determine the best strategy to increase efficacy by modulating treatment sequences.
